# The effect of midazolam on pain control after knee arthroscopy: a systematic review and meta-analysis

**DOI:** 10.1186/s13018-017-0682-0

**Published:** 2017-11-21

**Authors:** Xiaojun Chen, Xiaoqing Mou, Zhiyu He, Yong Zhu

**Affiliations:** 1 Department of Orthopaedics, the Affiliated Traditional Chinese Medicine Hospital of Southwest Medical University, Luzhou, Sichuan China; 2 Department of Radiology, the Affiliated Hospital of Southwest Medical University, Luzhou, Sichuan China; 3 Department of Orthopaedics, the First Affiliated Hospital of Chongqing medical university, No. 1 Yi Xue Yuan Road, Yuzhong District, Chongqing, 400016 China

**Keywords:** Midazolam, Pain control, Knee arthroscopy, Pain scores, Meta-analysis

## Abstract

**Background:**

Midazolam has some potential in pain control of patients undergoing knee arthroscopy. However, the results remain controversial. We conduct a systematic review and meta-analysis to explore the effect of midazolam on pain control after knee arthroscopy.

**Methods:**

PubMed, EMbase, Web of science, EBSCO, and Cochrane library databases are systematically searched. Randomized controlled trials (RCTs) assessing the effect of midazolam on pain management after knee arthroscopy are included. Two investigators have independently searched articles, extracted the data, and assessed the quality of the included studies. This meta-analysis is performed using the random-effect model.

**Results:**

Six RCTs are included in this meta-analysis. Compared with control intervention after knee arthroscopy, midazolam intervention can significantly reduce the pain scores (standard mean difference (Std. MD) = − 3.70; 95% confidence interval (CI) = − 6.81 to − 0.60; *P* = 0.02), the number of patients requiring analgesics (risk ratio (RR) = 0.66; 95% CI = 0.49 to 0.88; *P* = 0.005), and analgesic consumption (Std. MD = −1.62; 95% CI = − 3.04 to − 0.19; *P* = 0.03), as well as increase the time to first analgesic requirement (Std. MD = 1.58; 95% CI = 0.17 to 2.99; *P* = 0.03). In addition, midazolam intervention results in no increase in adverse events following knee arthroscopy (RR = 0.74; 95% CI = 0.18 to 2.98; *P* = 0.67).

**Conclusions:**

Midazolam intervention is revealed to substantially reduce the pain scores, the number of patients requiring analgesics, and analgesic consumption, as well as improve the time to first analgesic requirement after knee arthroscopy.

## Background

Postoperative pain management is an important and challenging matter to improve patients’ comfort, daily activity, satisfaction, and early hospital discharge [1–5]. Knee arthroscopy has become very common and causes moderate to severe pain after the surgery [3, 6, 7]. Different drugs and methods have been developed to provide the effective, safe, and long-lasting approaches for pain control after arthroscopic knee surgery. The main methods include systemic medication, peripheral or central blocks, and intraarticular drug administration [8–11]. For example, the intraarticular route is one of the analgesic approaches for pain management after knee arthroscopy [12], and some studies have reported the efficacy of midazolam, tramadol, bupivacaine, dexmedetomidine, morphine, and etoricoxib for the pain management of knee arthroscopy [12–16].

Midazolam is one of the clinically water-soluble benzodiazepines and effective to produce the analgesic effect through the neuraxial pathways [17–19]. The organs and joints of humans have the benzodiazepine receptor, and midazolam is revealed to produce the analgesic effect through the gamma-aminobutyric acid receptor in the spinal cord [20–22]. Previous studies have reported that the midazolam (75 μg/kg) through the intraarticular route can decrease the pain intensity for arthroscopic knee surgery [13]. In addition, the intrathecal midazolam (2 mg) is reported to prolong the duration of analgesia without any adverse effects following knee arthroscopies [23].

However, some relevant RCTs have shown that midazolam has no remarkable influence on pain control, the time to first analgesic requirement after the knee arthroscopy [1, 24, 25]. Considering these inconsistent effects, we therefore conduct a systematic review and meta-analysis of RCTs to evaluate the effectiveness of midazolam intervention on pain management in patients undergoing knee arthroscopy.

## Materials and methods

This systematic review and meta-analysis are conducted according to the guidance of the Preferred Reporting Items for Systematic Reviews and Meta-Analysis statement [26] and the *Cochrane Handbook for Systematic Reviews of Interventions* [27]. No ethical approval and patient consent are required, because all analyses are based on previous published studies.

### Literature search and selection criteria

PubMed, EMbase, Web of science, EBSCO, and the Cochrane library are systematically searched from inception to August 2017, with the following keywords: midazolam and knee arthroscopy. To include additional eligible studies, the reference lists of retrieved studies and relevant reviews are also hand-searched and the process above is performed repeatedly until no further article is identified.

The inclusion criteria are as follows: (1) the study population are patients undergoing knee arthroscopy; (2) the intervention treatments are midazolam intervention versus placebo; and (3) the study design is RCT.

### Data extraction and outcome measures

The following information is extracted from the included RCTs: first author, publication year, sample size, baseline characteristics of patients, midazolam, control, study design, pain scores, the time to first analgesic requirement, the number of patients requiring analgesics, analgesic consumption, and adverse events. The author would be contacted to acquire the data if necessary.

The primary outcome is pain scores. Secondary outcomes include the time to first analgesic requirement, the number of patients requiring analgesics, analgesic consumption, and adverse events.

### Quality assessment in individual studies

The Jadad Scale is used to evaluate the methodological quality of each RCT in this meta-analysis [28]. This scale consists of three evaluation elements: randomization (0–2 points), blinding (0–2 points), and dropouts and withdrawals (0–1 points). One point would be allocated to each element if they have been mentioned in article, and another one point would be given if the methods of randomization and/or blinding had been appropriately described. If the methods of randomization and/or blinding are inappropriate, or dropouts and withdrawals have not been recorded, one point is deducted. The score of Jadad Scale varies from 0 to 5 points. An article with Jadad score ≤ 2 is considered to be of low quality. If the Jadad score is ≥ 3, the study is thought to be of high quality [29].

### Statistical analysis

Std. MD with 95% CI for continuous outcomes (pain scores, the time to first analgesic requirement, analgesic consumption) and RR with 95% CI for dichotomous outcomes (number of patients requiring analgesics and adverse events) are used to estimate the pooled effects. All meta-analyses are performed using the random-effects model with DerSimonian and Laird weights. Heterogeneity is tested using the Cochran Q statistic (*p* < 0.1) and quantified with the *I* ^2^ statistic, which describes the variation of effect size that is attributable to heterogeneity across studies. An *I* ^2^ value greater than 50% indicates the significant heterogeneity. Sensitivity analysis is performed to detect the influence of a single study on the overall estimate via omitting one study in turn when necessary. Owing to the limited number (< 10) of the included studies, publication bias is not assessed. *P* < 0.05 in two-tailed tests is considered statistically significant. All statistical analyses are performed using Review Manager Version 5.3 (The Cochrane Collaboration, Software Update, Oxford, UK).

## Results

### Literature search, study characteristics, and quality assessment

**Fig. 1:**
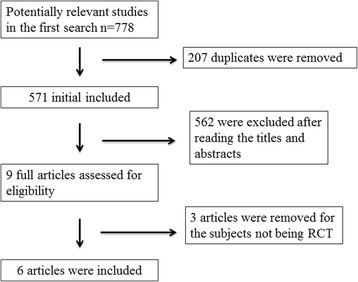
Flow diagram of study searching and selection process

The flow chart of selection process and detailed identification is presented in Fig. 1. Seven hundred seventy-eight publications are identified through the initial search of databases. Ultimately, six RCTs are included in the meta-analysis [1, 13, 23–25, 30]. The baseline characteristics of six eligible RCTs in the meta-analysis are summarized in Table 1. The six studies are published between 2008 and 2014, and sample sizes range from 30 to 70. The methods of midazolam application include intraarticular, intravenous, and intrathecal approaches. Three RCTs report 75 μg/kg intraarticular injection of midazolam [13, 25, 30], and three RCTs report midazolam as adjunctive therapy to intrathecal hyperbaric bupivacaine (0.5%) [23], intravenous ketamine (0.15 mg/kg) [1], and intraarticular lidocaine (2%) [24].

**Table 1:** Characteristics of included studies

No.	Author	Midazolam group						Control group						Jadad scores
		Number	Age (years)	Male (*n*)	Body mass (kg)	Surgery duration (min)	Methods	Number	Age (years)	Male (*n*)	Body mass (kg)	Surgery duration (min)	Methods	
1	Sajedi 2014	25	26.9 ± 5.4	19	71.9 ± 4.5	49.2 ± 4.3	75 μg/kg intraarticular injection of midazolam and 10 ml intravenous injection of isotonic saline	25	27.5 ± 5.1	22	70.7 ± 4.5	49.2 ± 4.6	Intraarticular and intravenous injection of isotonic saline	5
2	Nanjegowda 2011	25	32.48 ± 11.13	9	63.36 ± 11.79	–	0.5% hyperbaric bupivacaine with preservative-free midazolam 2 mg intrathecally	25	35.24 ± 10.91	10	63.36 ± 11.79	–	0.5% hyperbaric bupivacaine with saline intrathecally	3
3	He 2010	15	23–34	–	46.7–62.5	–	75 μg/kg intraarticular injection of midazolam	15	23–34	–	46.7–62.5	–	Intraarticular injection of saline	3
4	Cagla 2009	20	18–57	9	50–110	–	Ketamine 0.15 mg/kg and midazolam 0.01 mg/kg intravenously	20	16–65	11	55–95	–	Intravenous ketamine 0.15 mg/kg	4
5	LI 2008	35	18–65	–	–	–	Intraarticular 2% lidocaine and 2 mg midazolam	35	18–65	–	–	–	Intraarticular 2% lidocaine	3
6	Batra 2008	20	41 ± 4	14	69 ± 4	–	Intraarticular midazolam 75 μg/kg	20	40 ± 4.6	13	75 ± 3	–	Intraarticular saline	4

Among the six RCTs, three studies report the pain scores [23–25], four studies report the time to first analgesic requirement [1, 13, 23, 30], two studies report the number of patients requiring analgesics [25, 30], three studies report the analgesic consumption [1, 13, 30], and four studies report the adverse events [13, 23, 25, 30]. Jadad scores of the six included studies vary from 3 to 5, and all six studies are considered to be high-quality ones according to quality assessment.

### Primary outcome: pain scores

**Fig. 2:**
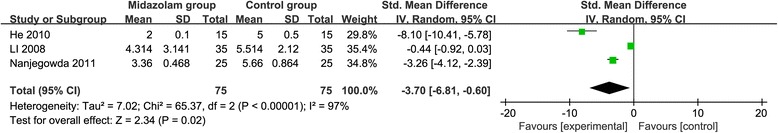
Forest plot for the meta-analysis of the pain scores

This outcome data is analyzed with the random-effects model, and the pooled estimate of the three included RCTs suggest that compared to the control group after knee arthroscopy, midazolam intervention is associated with significantly decreased pain scores (Std. MD = −3.70; 95% CI = − 6.81 to − 0.60; *P* = 0.02), with significant heterogeneity among the studies (*I* ^2^ = 97%, heterogeneity *P* < 0.00001) (Fig. 2).

### Sensitivity analysis

Significant heterogeneity is observed among the included studies for the primary outcome. Thus, we perform the sensitivity analysis by omitting one study in each turn and perform subgroup analysis based on different approaches of midazolam to detect the source of heterogeneity, but there is still significant heterogeneity.

### Secondary outcomes

**Fig. 3:**
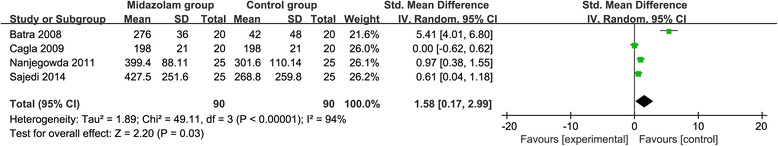
Forest plot for the meta-analysis of the time to first analgesic requirement (min)

**Fig. 4:**
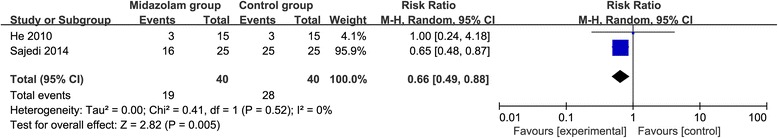
Forest plot for the meta-analysis of the number of patients requiring analgesics

**Fig. 5:**
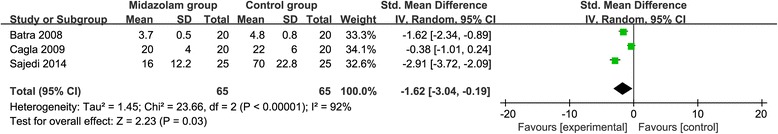
Forest plot for the meta-analysis of analgesic consumption (mg)

**Fig. 6:**
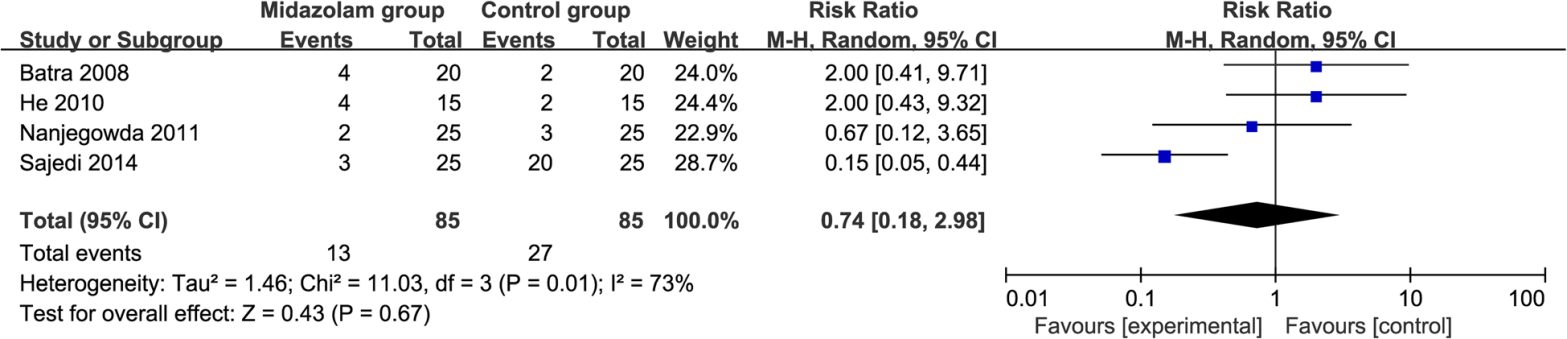
Forest plot for the meta-analysis of adverse events

Compared with control intervention following knee arthroscopy, midazolam intervention results in significantly prolonged time to first analgesic requirement (Std. MD = 1.58; 95% CI = 0.17 to 2.99; *P* = 0.03; Fig. 3), reduced number of patients requiring analgesics (RR = 0.66; 95% CI = 0.49 to 0.88; *P* = 0.005; Fig. 4), and analgesic consumption (Std. MD = − 1.62; 95% CI = − 3.04 to − 0.19; *P* = 0.03; Fig. 5). There is no increase in adverse events after midazolam application (RR = 0.74; 95% CI = 0.18 to 2.98; *P* = 0.67; Fig. 6).

## Discussion

Arthroscopic surgery can cause considerable postoperative pain because of the irritation of free nerve endings of synovial tissue, anterior fat pad, and joint capsule [31–33]. Our meta-analysis suggests that compared to control intervention for knee arthroscopy, midazolam intervention substantially decreases the pain scores, the number of patients requiring analgesics, and analgesic consumption, as well as increases the time to first analgesic requirement, with no increase in adverse events. To our knowledge, this is the first meta-analysis to assess the influence of midazolam on pain control in patients with knee arthroscopy.

Regarding the sensitivity analysis, there is still significant heterogeneity by omitting one study in each turn and performing subgroup analysis on intraarticular or intrathecal approaches. Two factors may account for this significant heterogeneity. Firstly, different routes such as intraarticular or intrathecal approaches of midazolam application have different influence on pain management after knee arthroscopy. Secondly, there may be remarkable difference of pain management between pure midazolam and its adjunctive therapy to other analgesics. For instance, midazolam supplementation can reduce pain severity better than intraarticular 2% lidocaine, but there is no significant difference [24]. In contrast, intraarticular injection of midazolam can significantly decrease the pain scores compared to intraarticular injection of saline [25].

Previous studies have demonstrated that intraarticular injection of midazolam is able to reduce postoperative pain, sedation scores, and total postoperative analgesic consumption and delay the time of first analgesic administration compared to intravenous midazolam following knee arthroscopy, possibly because the analgesic effect of intraarticular midazolam may mainly act at the peripheral site in the joint, but this effect is less effective through systemic administration [30]. Consistently, intraarticular administration of tramadol is reported to produce longer duration of analgesia and lower pain scores and analgesic consumption than intravenous administration of tramadol after knee arthroscopy [34]. In addition, one included RCT has reported that intraarticular administration of midazolam 50 and 75 μg/kg can decrease postoperative pain and delay analgesic requirement compared to placebo group, but there is no dose-dependent effect with the administration of midazolam 50 or 75 μg/kg [13].

Several limitations should be taken into account. Firstly, our analysis is based on six RCTs but all of them have a relatively small sample size (*n* < 100). Overestimation of the treatment effect is more likely in smaller trials compared with larger samples. The doses and methods of midazolam administration in the included studies are different, and they probably affect the pooled results. Next, the optimal dose and approach of midazolam application for knee arthroscopy remains elusive. Finally, some unpublished and missing data may lead the bias to the pooled effect.

## Conclusion

Midazolam administration shows some ability to reduce pain severity in patients undergoing knee arthroscopy and should be recommended to be administrated in these patients with caution.

## Abbreviations

CI: Confidence interval
RCTs: Randomized controlled trials
RR: Risk ratio
Std. MD: Standard mean difference
